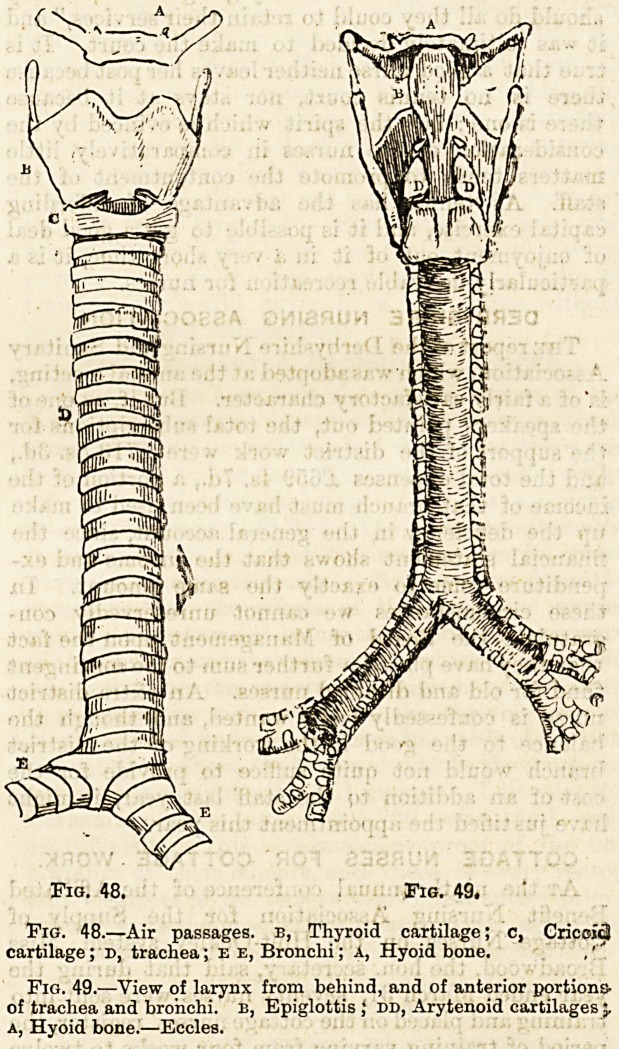# The Hospital. Nursing Section

**Published:** 1902-06-28

**Authors:** 


### The Hospital.

### Nursing Section.

Contributions for this Section of "The Hospital" should be addressed to the Editor, "The Hospital" Nursing Section, 28 & 29 Southampton Street, Strand, London, W.C.

No. 822.—Vol. XXXII. *SATURDAY, JUNE* 28, 1902.

### Notes on News from the Nursing World.

### THE ROYAL FUNCTION AT GUY'S HOSPITAL.

The arrangements for the opening of the Henrietta Raphael Nurses' Home by the Prince and Princess of Wales on Monday, July 7, are making good progress. We understand that the formal ceremony will take place in a large pavilion to be erected in the park, with the Home itself as a background. This pavilion is to be as large as circumstances will allow, but even then it will only accommodate a proportion of the guests. It may save trouble and disappointment if we state that everyone, whether a Governor of Guy's or a member of the staff, is expected to be the holder of a card of admission.

### THE MATRONSHIP OF ST. THOMAS'S HOSPITAL.

Our intimation last week of the resignation by Miss L. M. Gordon of the office of matron of St. Thomas's Hospital, has naturally provoked a flutter of excitement in the nursing world, and there is much speculation as to the manner in which the vacancy will be fillled. The choice of a successor to Miss Gordon rests with the House Committee, and the Governors have decided not to advertise. The great importance of the duties, the high standing of the Nightingale school, and the opportunities which whoever is elected to the post will be afforded of influencing the future of nursing, invest the appointment with many attractions. Miss Gordon, although retiring from hospital work, will continue to hold her position on the Advisory Board of Queen Alexandra's Imperial Military Nursing Service, and her valuable experience will therefore be still, in a measure, available for the benefit of nurses.

### "A MIGHTY POWER FOR GOOD."

An extremely pleasant feature of the nineteenth annual meeting of the Holywood Nursing Society was that it represented all shades of religious thought. Even in Ireland the nursing movement breaks down the barriers which so often bar the way to concerted action in the interests of humanity on the part of the churches. At the Holywood meeting Canon Duke, in proposing the adoption of the report, said that looking round on the company he thought how appropriate was the text, "How good it is for brethren to dwell in unity." The remark was well-timed, for the Lord Bishop of Ossory presided over the gathering, and Roman priests and Nonconformist ministers vied with Anglican clergy in their praise of the organisation. The chairman also struck the right note when, speaking after Canon Duke, he observed that "he knew of nothing that had done so much good to the country and humanity as those societies, which had dug into that great mine of intelligent power that had practically lain untouched until the days of Florence Nightingale." He pointed out that Miss Nightingale and those who worked so hard in the Crimean War got the people to see what a mighty power there was for good in "nursing sisters," and that the movement begun on the battlefields extended, by degrees, to the hospitals, the homes of the well-to-do, and finally, by means of nursing organisations, to the houses of the sick poor. In Holywood the people show their practical appreciation of the work by supporting their own association in such a manner that at the end of the last year there was a balance of £76 in hand. The benefit derived from it by the poor is very great and it is not the least of its recommendations that it has drawn persons of opposing views on many points together and scattered sectarian feeling.

### THE UNTRAINED "LADY SUPERINTENDENT."

The author of the thoughtful article on "The Position of a Private Nurse," which appears in another part of the paper, is exclusively responsible for the opinions she expresses. The same remark applies, of course, to the letter of "H.," who advocates the supervision of private nursing homes by Government. But both the questions dealt with are of profound interest, and will bear a good deal of discussion. The writer of the article lays her hand on a point of vital importance both to the individual private nurse and also to the inmates of private nursing homes. "Too often," she says, "the lady superintendents of private nursing homes are quite untrained women." This is true, and we have no doubt whatever that it is the main cause for the existence of nursing homes of the character described by "H." An untrained matron cannot, as our contributor contends, possibly judge of the training of her nurses. "Ignorant herself of the necessary discipline of a nurse's life, she cannot instil discipline into those she superintends." The results are necessarily disastrous. Untrained superintendents often engage untrained nurses, and the whole system upon which the home is based is thus utterly bad. But there is a more simple remedy than the establishment of some vast new organisation for nurses. It lies chiefly with the public, who should not assume that because a house is called a nursing home it merits that title. No one should obtain a nurse from one of those institutions, or allow a sick friend to enter one for treatment, without ascertaining the nature of the credentials of the lady superintendent. If she be unwilling or unable to give them, the public should have nothing to do with her; and if she produces credentials of training which are false, she is liable to be prosecuted as an impostor. The untrained lady superintendent, who is often no more a lady than she is a nurse, is the bane of private nursing homes, and her total abolition would at least be one step in the direction of improvement in the condition of private nurses.

### THE AGE QUESTION.

It will have been noticed that the matron of the London Homœopathic Hospital told our Commissioner last week that in choosing probationers she does not attach "great importance" to the question of age. According to the regulations, no one can be admitted who is under 22 nor over 30, but the matron does not refrain from recommending a candidate for admission who either has not reached, or has passed, the prescribed age, if she seems particularly suitable. The matron's view is that the personality of the woman is more important than her age, and we entirely agree with her. There must, however, be rules as to age, and generally speaking it is wise to adhere to them. But, while it is often convenient to be able to decline to accept a candidate because she is a little beyond the regulation age, it is a pity that a hospital should lose the services of a woman who is obviously admirably cut out for the work because she confesses that she is a year or two over the age of admission.

### NURSES IN THE HOP-YARD.

For the past two years a lady at Malvern has run a tea and refreshment tent in one of the hop-yards of the neighbourhood during the hop-picking season. She has been assisted by a hospital nurse, and has found the services of the latter so greatly appreciated by the workers, who, if anything was the matter, flew at once to "nurse" in the tent, that she is anxious that nurses should volunteer for the work in the coming autumn. In fact, she believes that there should be a trained nurse in every hop-yard. We do not doubt the usefulness of this sphere of labour; but with regard to the idea of volunteers, it is not advisable that nurses should devote [any of their holidays to work which, though to some extent different, bears far too much resemblance to their ordinary duties to render it desirable. It appears that in Worcestershire the refreshment part of the undertaking can be made to pay its own way, and that the expenses of the board and lodging of the lady workers are defrayed by subscription. But they are not remunerated. Nurses who can afford to give time, and who are not in active service may be glad to avail themselves of an opportunity of helping an excellent cause.

### THE FIRST CERTIFICATE AT WAKEFIELD WORKHOUSE INFIRMARY.

At the conclusion of the last meeting of the Infirmary Committee of the Wakefield Guardians, Miss Quinn was presented by the chairman with a first-class certificate, having passed her examination at the end of a three years' course of training. In addition to the members of the committee, there were also present the infirmary superintendent, the medical officer, the master, and the acting clerk. The chairman said it was with great pleasure that he presented this, the first certificate gained at the infirmary. He hoped that the rest of the probationers would persevere as Nurse Quinn had done, and that her succcess would be an incentive to them to pay every attention to their lectures and studies. Nurse Quinn thanked the chairman and committee for their kindness and encouragement, and also spoke of the help she had received during her training from the medical officer and the superintendent nurse, Miss Lightowler. The chairman of the board in congratulating Miss Quinn, said he was glad that the difficulty which at first appeared to meet the committee had been overcome, and that nurses were enabled to obtain the certificates in such a well-equipped infirmary.

### THE TRUE NURSING SPIRIT.

The public distribution of prizes to the probationers of the East Suffolk Hospital who stood highest in marks as the result of the examination was for many reasons an interesting event. The chairman, Mr. F. W. Mason, said that at the hospital there were at present some 23 sisters and nurses, of whom 15 were undergoing their training extending over three years, a period which, he contended, was none too long for a nurse to serve to become efficient. Of the fifteen in question eight were in their third year, and the results of the examination were very creditable. The prize winners who received their prizes—which were either medical or nursing books—from Mrs. Cobbold were Nurses Jay, Morland, and Brown. Mrs. Cobbold, having made the presentation, delivered an excellent address, in the course of which she said that nursing was a noble profession, if it were entered into in the right spirit. "Did the nurses of the East Suffolk Hospital enter with a spirit such as that possessed by Miss Nightingale, whose services were invaluable on the battlefield where hospitals did not exist, or were they entering it as a profession which would simply keep them, and where their minds were not involved in the work for the sick and injured?" She entreated any who regarded it from the latter point of view to give up nursing, because she was confident that, in spite of training, one would never be a real nurse without love, or without self-sacrifice.

### THE NEWCASTLE CATHEDRAL NURSES.

We regret to hear from the annual report of the Newcastle Cathedral Nurse and Loan Society, which was adopted at the meeting last week, that there is a deficiency of no less than £255 on the year's working, the total receipts being £1,587, as against a total expenditure of £1,842. The chairman attributes it largely to the fact that the society does its great work "so unostentatiously." But, however desirable it may be that the efforts of the individual nurses should not be paraded before the public, the existence of the society and the objects for which it is carried on, cannot be too widely proclaimed. The more thoroughly the rich people in the thriving city of Newcastle and its environs, know the needs of the sick poor at their doors, the better should be the chance of enlisting their sympathy and support. Last year the Cathedral nurses attended 1,756 cases, and paid 17,152 visits. This is a sufficient proof of the importance of their labours, and there is every reason why members of the society who collect subscriptions should make the most of the figures, which attest the need of placing it on a sound financial footing.

### THE QUEEN VICTORIA INSTITUTION AT WOLVERHAMPTON.

Even at Wolverhampton, where the Queen Victoria Nursing Institution is admirably managed, the committee find it difficult to combine the nursing of paying patients with that of nursing the sick poor, and the lack of funds for carrying on the district nurses' home, "especially in the way of increased annual subscriptions," is, we regret to learn, causing them much anxiety. There is no question as to the value of the work done by the district branch. Last year the number of cases attended by the district nurses was 884, but seeing that the fees paid by patients are known to amount to a considerable sum, the public may to some extent be under the impression that the institution itself is largely, if not entirely, self-supporting. It is, we fear, inevitable that in such cases there should be people who will not take the trouble to discriminate between the two branches of the organisation. On the other hand, that the paying branch is highly appreciated in Wolverhampton is shown by the fact that the nurses attached to it earned in the year £118 in bonuses.

### TENNIS FOR NURSES.

At a quarterly meeting of the Hanley, Stoke and Fenton Joint Hospital Board the subject of a tennis ground for the nurses was discussed. It had been mentioned before, but had been allowed to remain in abeyance. The chairman, however, thought that with the advent of summer action should be taken. His view was "that when they got good nurses they should do all they could to retain their services," and it was ultimately decided to make the court. It is true that a good nurse neither leaves her post because there is no tennis court, nor stays at it because there is one; but the spirit which is evinced by the consideration of the nurses in comparatively little matters tends to promote the contentment of the staff. As tennis has the advantage of affording capital exercise, and it is possible to get a good deal of enjoyment out of it in a very short time, it is a particularly desirable recreation for nurses.

### DERBYSHIRE NURSING ASSOCIATION.

The report of the Derbyshire Nursing and Sanitary Association, which was adopted at the annual meeting, is of a fairly satisfactory character. But if, as one of the speakers pointed out, the total subscriptions for the support of the district work were £713 8s. 3d., and the total expenses £659 4s. 7d., a portion of the income of that branch must have been used to make up the deficiency in the general account, since the financial statement shows that the income and expenditure come to exactly the same amount. In these circumstances we cannot unreservedly congratulate the Board of Management upon the fact that they have placed a further sum to the contingent fund for old and disabled nurses. An extra district nurse is confessedly badly wanted, and though the balance to the good in the working of the district branch would not quite suffice to provide for the cost of an addition to the staff last year, it might have justified the appointment this year.

### COTTAGE NURSES FOR COTTAGE WORK.

At the ninth annual conference of the Affiliated Benefit Nursing Association for the Supply of Cottage Nurses on the Holt-Ockley system, Miss Broadwood, the hon. secretary, said that during the year ended March 31, seventy nurses were sent into training and placed on the cottage nurses' register, the period of training varying from four weeks to twelve months. Miss Broadwood, responding to the challenge that a higher training is desirable, takes the negative view, and claims in support of it the fact that those who are placed on the register "at once obtain positions as district nurses or assistant nurses in small hospitals, or large workhouse infirmaries." We do not doubt Miss Broadwood's desire to be accurate, but it is certain that some of the small hospitals, and most of the separate poor-law infirmaries, will now only have fully-trained nurses on their staff. In any case, cottage nurses should be confined to cottage work.

### VICTORIA HOSPITAL FOR CHILDREN.

An open-air fête in aid of the Victoria Hospital for Children is to be held on July 2nd, in the Ranelagh Gardens, adjoining the Royal Hospital, Queen's Road, Chelsea. Princess Louise, Duchess of Argyll, will lay the foundation-stone of the new buildings at 2 o'clock, and afterwards preside at the flower stall. The Countess Carrington will sell toys, and Viscountess Chelsea, assisted by her sister, the Hon. Mrs. Hardinge, and Mrs. Martin Smith, pottery. The "sweets" stall will be managed by Lady Audrey Buller, and "cigarettes" by the Hon. Mrs. Eustace Fitzgerald, assisted by Viscountess Barrington and Miss Fitzgerald. The hospital stall is under the charge of the Hon. Mrs. R. A. Lubbock and Miss Watson, the matron, assisted by the sisters and nurses of the hospital.

### A GOOD BEGINNING AT FLINT.

At the first annual meeting of the Flint District Nursing Association it was stated that the receipts amounted to £90 18s. 2d. and the disbursements to £56 13s. 4d. The nurse attended 102 cases and paid 3,079 visits equal to an average of ten visits daily. This is a good beginning from every point of view, and we endorse the hope expressed by the Mayor, and by Dr. J. Humphry Williams, that the County Council will see their way to continue to support the Association.

### WEST EROMWICH NURSES.

In connection with the recnt opening of the Nurses' Home at West Bromwich we are asked to state that the nursing staff now consists of a trained superintendent, three fully trained nurses, and a one-year's probationer; the superintendent and the senior nurse having been for many years on the staff of the North London District Nursing Association. The Home provides accommodation for eight nurses and one matron, and should therefore suffice to meet the requirements of the town for some time to come.

### A DEFICIENCY AT HARROGATE.

It is not creditable to Harrogate that the sixth annual report read at the meeting this month shows a deficiency of over £5 on the year's working, and in the circumstances the subscribers, perhaps wisely, adopted a proposal to alter the constitution of the society and place it on different lines. At the instance of Mrs. Morrison a ladies' committee, with an advisory board of gentlemen, will in future be responsible for the management. There may not be a very large amount of nursing to be done in Harrogate, but that is no excuse for the inadequate support given by the residents to the society. Moreover, there are the sick visitors, who derive health from the famous springs. Between the two it should be a very easy matter to raise such a small sum for the benefit of the poor as £225 a year.

### SHORT ITEMS.

The sum contributed by Bournemouth to the Women's Memorial to Queen Victoria is £700, or only £2 less than Bristol, which has sent the largest amount from any provincial borough to the fund.

### Lectures to Nurses on Anatomy.

By W. Johnson Smith, F.R.C.S., Principal Medical Officer, Seamen's Hospital, Greenwich.

### LECTURE XX.—THE ORGANS OF RESPIRATION.

In the lecture on the organs of circulation it was pointed out that the blood in its course from the right to the left side of the heart passed through the lungs and was there purified, being changed from a dark purple to a bright red fluid—from venous to arterial blood. This purifying process which exists chiefly in an exchange of poisonous

Fig. 47.—The lungs; the front portion of the left organ removed to show branching of the left branches. A, Trachea.

carbonic acid gas (carbon dioxide) contained in the venous blood for vitalising oxygen supplied by the atmospheric air, necessitates on the one hand a free and continuous flow of blood through the lungs, and on the other hand, an uninterrupted supply and a frequent and regular renewal of fresh air. The flow of the blood, we have learnt, is kept up by the constant and rhythmical movements of the heart. The corresponding supply of air depends on the natural performance of the function known as respiration or breathing, in which the chest is expanded and contracted from 16 to 24 times per minute.

The organs of respiration are the *air passages* through which fresh air is admitted into and impure air is discharged from the chest, and the *two lungs* in which the inspired air is brought into close relation with the circulating venous blood.

The apparatus for the admission and discharge of air consists of a long elastic tube—the *trachea*, or windpipe—situated in front of the neck, which is surmounted by a box of cartilage—the organ of voice—known as the *larynx*, and terminating below in a dividing and subdividing series of tubes (bronchi, bronchial branches, bronchial tubes) which are distributed to all parts of the lungs, and which by their ultimate and most minute branches form a considerable portion of the structure of these organs. (Fig. 47).

The framework of the larynx is formed by two stiff though light cases of cartilaginous structure known as the *thyroid* and the *cricoid* cartilages, both of which can be felt beneath the skin in the middle line of the neck and are important guides in operations for opening the windpipe. (Fig. 48, B, C). The thyroid cartilage, which is much larger and more prominent than the cricoid, consists of two lateral plates joined together in front and diverging outwards and backwards so as to leave a large gap behind. The upper margin is deeply notched in the middle line and forms a distinct protrusion, termed the *pomum Adami*, which is more prominent in the male than in the female. From the upper and lower angles of each plate behind project two curved processes of cartilage called the *cornua*, or horns of the thyroid. The cricoid cartilage is a complete ring which is much deeper and thicker behind than in front, resembling in this respect a signet ring. The space between the narrow portion of the ring in front and the lower margin of the thyroid cartilages is filled in by an elastic membrane called the crico-thyroid membrane. Attached to the back part of the anterior angle of the thyroid cartilage is a large leaf-shaped plate of cartilage—the *epiglottis* (fig. 49 B)—the upper and free margin of which may sometimes be seen on forcibly depressing the back part of the tongue and which, on retraction of the tongue and during swallowing, is pressed backwards and downwards so as to cover the entrance to the larynx.

The large space enclosed by the thyroid and cricoid cartilages is almost completely filled up by small muscles and folds of mucous membrane, and the passage to the windpipe is reduced to a narrow chink—the *rima glottidis*—between two elastic ligaments which extend from the angle of the

Fig. 48.

Fig. 49.

Fig. 48.—Air passages. B, Thyroid cartilage; C, Cricoid cartilage; D, trachea; E E, Bronchi; A, Hyoid bone.

Fig. 49.—View of larynx from behind, and of anterior portions of trachea and bronchi. B, Epiglottis; DD, Arytenoid cartilages; A, Hyoid bone.—Eccles.

thyroid cartilage backwards with a slight divergence to two small movable cartilages called the *arytenoid* cartilages (fig. 49, D) attached to the upper margin of the cricoid. These ligaments are the *true vocal cords*, so called in contra-distinction to two folds of mucous membrane at a higher level which, as they take no part in the production of sound, are called the *false vocal cords*. The rima glottidis is widened during the inspiration of air and closes during

### LECTURES TO NURSES ON ANATOMY.—*Continued.*

expiration. The length of the true vocal cords differs in the two sexes and in young and elderly subjects. They are relatively short in females and boys, and longer in men. As a shrill tone of voice corresponds to a short cord, and a deep tone to a long cord, we may thus account for the difference between the female and male voices and for the so-called "breaking" of the voice in boys during the increase in the size of the thyroid cartilage that occurs at the beginning of puberty.

If we bear in mind how narrow the entrance to the wind-pipe is, we may readily conceive how the presence of even a small foreign body, as a bead or pea over the vocal cords, or swelling of the surrounding mucous membrane may block the passage and cause death by suffocation.

The air reaches the larynx by way of the mouth and nostrils and a large cavity at the back of the mouth called the *pharynx*, which communicates below with the air passages in front and with the gullet or œsophagus behind. In swallowing, therefore, the food before it enters the gullet must pass across the inlet of the larynx. Under ordinary conditions the admission of even small portions of food is prevented by retraction of the tongue and depression over the epiglottis over the top of the larynx, but in hasty and careless swallowing during a deep inspiration, or in an attempt to feed an unconscious patient, part of the food may "go the wrong way," and set up the serious symptoms resulting from the presence of a "foreign body in the air-passages."

The *trachea* (figs. 48, D) is an open and elastic tube a little more than four inches in length and a little less than one inch from side to side. Its patency is due to curved bands of stiff cartilage, from 15 to 20 in number, and its elasticity to intervening elastic membrane. If we look at a transverse section of this large air-tube we find that it does not form a perfect circle, and that whilst it is curved in front and at the sides it is flattened behind where it is in contact with the front of the gullet. The cartilaginous hoops or, as they are usually called, the rings of the trachea, are not perfect rings, as along the posterior surface of the tube about one-third of each is missing. Some of the rings are single strips of cartilage, others are joined together by a narrow intervening band.

The first ring is connected with the cricoid by a layer of elastic fibrous tissue similar to that between each pair of rings. The last ring is Y-shaped, the angle on each side corresponding to the junction of one of the two bronchi. The junction of the trachea with the two bronchi—the bifurcation of the trachea as it is called—is behind the sternum, and about one inch below the notch on the upper margin of this bone.

The front of the trachea—the second and two following rings—is covered by a band of soft glandular tissue forming a bridge or isthmus between the two lateral lobes of a large gland situated in front of the neck. This structure, called the *thyroid gland*, is barely perceptible in health, but in certain states of disease becomes much enlarged, presenting the different forms of goitre. This enlargement is most marked in the sad condition of mental and bodily degeneration known as cretinism, so prevalent in Switzerland and other mountainous countries. In young children there is another gland of similar structure—the thymus gland—in front of the lower part of the trachea and behind the top of the breast bone.

In cases of obstruction of the larynx, and of blocking of the lower air-passages by the so-called false membrane of diphtheria, or by a foreign body, it is found necessary to admit air and to give relief by opening the trachea—performing the operation of tracheotomy. This operation which in many cases, and especially with young children, is one of great difficulty on account of bleeding and the rapid movements of the trachea is usually performed just below the cricoid and above the isthmus of the thyroid gland.

### The Position of a Private Nurse.

BY AN OCCASIONAL CONTRIBUTOR.

IT is not my purpose in this short paper to give a history of the rise and fall of private nursing, though I feel it would be most useful information to many of my readers. The great body of workers who call themselves private nurses is altogether so scattered and disjointed that the world hardly looks upon it as a distinct organisation at all, and perhaps it may be this want of help and recognition from its fellows which has to do with its weakness and, as I think, it's sad falling away from a most excellent beginning.

This fact is too patent for me to attempt to deny it, but there are certain points connected with it which I think it would be well to consider: First, whose fault is it, and secondly, what can be done to help? Of course all nursing, instead of being helped by the sublimity of its origin, appears at first sight to be hindered by it. This may sound very wrong, but there is truth in it nevertheless. Start a good work on a material foundation with a feeble effort, amid much struggling and scant encouragement, and if the purpose be true and the desire great the work grows, enthusiasm warms, and hearts glow in anticipation of a bright and successful future. Thus little by little the profession, organisation, enterprise, or whatever it may be gains in calibre, gathers weight and power, and the world gives it standing room, consenting at the same time to be benefited and ruled by it in its own particular path in the administration of affairs. But nursing started in all the splendour of the inspiration of saints, and great indeed has been its fall. The world recognised its utter lack of the commercial instinct, and it has been hampered at every turn. It was never meant to be a large trading enterprise; therefore it is hard, and well-nigh impossible, for a nurse with the calculating, anarchist type of mind of to-day to uphold the ideals with which she started and help to keep up the old-fashioned dignity of her calling.

### THE BEST NURSES WANTED.

Private nursing should be done only by the best nurses. A nurse who has never before known what it is to have no one in authority over her, to whom she may fly in an emergency, finds herself alone, maybe in the country, miles from a doctor. The patient's life may depend upon her competence to act promptly. She may find herself with a cantankerous old patient, who can be managed by no one who has not an immense amount of power of inspiring confidence. She has to know when it is necessary to summon medical aid, how to calm the nerves of anxious friends who would wear the doctor out with ceaseless calls upon his time and patience unless the nurse had enough knowledge and assurance to allay their fears. She must know how to walk into a disordered household and restore order out of chaos. Possibly, the head of the household has had a stroke: the family is distracted, the servants are weeping. A quiet, calm nurse, with a full knowledge of the gravity of the case, steps in, and order reigns again. She must, as far as her own self is concerned, be a cipher in the house where she is nursing, and it takes a well-disciplined and a good woman to accomplish that. She must realise that she enters a house to nurse back

### THE POSITION OF A PRIVATE NURSE.—*Continued.*

to health one who is sick. She must sink herself into oblivion, and be prepared to be just what that particular household requires her to be. There are many other qualifications necessary, but these seem to me the most salient, and when we look them over does it not seem ridiculous to expect all this of a half-trained woman, or of one who cannot succeed in other walks of her profession?

### The System at Fault.

In an institution a nurse goes all through her training under close supervision both as regards her work on duty and her mode of living off duty; with regard to her patients she gives no diet and applies no remedy that is not strictly under the doctor's orders. Should a patient suddenly develop severe symptoms, there are responsible people at hand who can at once instruct her how to act. In case of an operation, she has only to take her patient to the operating theatre, where all appliances are in perfect readiness. She requires, comparatively speaking, little mental power; she learns to obey, to live by rule, to go straight on with her daily duties. Even the expenditure of too ready sympathy is out of place in a hospital ward, though kindness and gentleness can never be overdone. Surely this all seems to point to the fact that there should be some extra training for private nurses; they should not bear all the brunt of their own deficiencies. Circumstances are against them, and they should be considered victims of a harmful and lax system.

### The Anarchists of the Profession who take up Private Nursing.

Frequently, too, they are the "failures" of the hospital and the infirmary, those who at the end of their probation are not efficient enough or morally good enough to be kept as staff or charge nurses; the anarchists of the profession, who will no longer brook authority, to whom rule and discipline are irksome and, so to speak, ridiculous; those who want freedom to work only when they choose, or when their exchequer demands it; those who want to earn more money than is possible in other branches of the profession; those who will not submit to the ordinary restrictions of hospital etiquette. Put these qualifications by the side of the requirements already mentioned, and the deplorable misfit of it all is obvious.

### A Word of Apology.

I cannot pass on without a word of apology to some of my fellow-workers—those who I know are leading a hard, unselfish life, isolated and yet much beloved, in many parts of the country. I have been a private nurse and lived in a home, and when I think of the devotion to duty and real efficiency of some few of those I lived amongst, I feel ashamed to be so severely criticising a branch of the profession to which they belonged. But it must be done, and some remedy for the present state of things must be found, and that promptly, to save this noble work from being dragged in the mire of unworthiness, when by right it should be classed as one of the first works of mercy in the land. Where shall we find a cure? It seems presumption for me to touch so large a subject, and in writing this paper I wanted merely to state the difficulties and ask others to suggest a remedy; but I cannot close without just a few suggestions about how it might be done, though I have no doubt there are many others who can work out the problem more fully than myself.

### A Few Suggestions.

First, can we not urge upon matrons of the larger training schools the immense responsibility devolving on them to more thoroughly weed out their candidates at the commencement of their career? It is hard to send young nurses away marked "unsuitable" just at the time, but far more really kind in the long run. Two or three months' trial shows the grit in a woman, and if only this weeding out could be done more carefully we should not find nurses in our ranks holding full certificates, and yet hopelessly inadequate for the profession to which they belong. Special provision should be made for training a private nurse for her own particular work. She should be taught responsibility, tact, sympathy, house management, sick cookery, reading aloud, neat serving of meals, etc.? All these are quite as necessary as a knowledge of anatomy or physiology, and yet as far as I know but few take the trouble to instruct their nurses in any one of them. It has been suggested that after hospital training at least three months might be spent in close attendance upon the sick in a nursing home, where there was no resident doctor, and where the superintendent made it her special duty to instruct the nurses in the real art of nursing in its very widest sense.

### The Untrained Lady Superintendent.

Then surely more care should be used in the appointments of lady superintendents of private nursing homes. So often they are quite untrained women. It seems a common occurrence, both in London and the provinces, for impecunious ladies with no qualifications whatever just to take a house and put a plate up to the effect that "Private nurses can be obtained at any hour," etc. The demand is at present so much greater than the supply that work is given in these nondescript houses to partially trained, or even to wholly untrained women, and the whole profession gets anathematised in consequence. An untrained matron cannot possibly judge of the training of her nurses. Ignorant herself of the necessary discipline of a nurse's life, she cannot instil discipline into those she superintends. She takes their fees or percentage, as the case may be, and has no further hold upon them, and one cannot help but pity them, for the pitfalls are many and they have no incentive to keep straight. Their whole life is a sort of forgery, they wear a uniform which is false, and, by virtue of their limited experience, they find themselves totally unable to cope with the tasks before them, and they are as it were walking on the edge of a precipice over which their own ignorance is continually threatening to push them.

### Co-operation and Protection Wanted.

They want co-operation and protection. Co-operation with the whole body of their fellow workers, and protection mainly from themselves and their own unworthy sisters. It may be rejoined that there are co-operations. Yes, so there are; but all of them of equal futility as far as this question is concerned. We want a bigger, a broader-minded co-operation—a sort of vast trades union (if I may call it so)—that will set this great work of nursing on the footing it needs and must have; a great union in which State hospitals, general hospitals, and all large institutions of the kind will combine, and help us nurses to be as efficient as we want to be; a union that will not recognise a partial training, but will demand a high standard, and will see that no nurse is engaged as a private nurse by any doctor anywhere until she has shown her credentials of efficiency; until it is known that she lives under the guidance of a thoroughly trained and competent superintendent to whom she is responsible, and who sees and cares that the nurse she turns out for this most important work is fully equipped for public service.

### Wants and Workers.

M. D. B. regrets that she cannot respond to all the 23 applications she has received for the portable Turkish bath. She hopes that the things will prove useful where they have been sent.

### Plants Suitable for Hospital Decoration and How to Tend Them.

BY AN IRISH CORRESPONDENT.

There is nothing so refreshing to the eye of the weary sufferer as he lies in bed as a plant growing strong and well, either flowering or otherwise; but the question naturally arises how may this health and strength of the plant be preserved when placed in the ward? It is taken for granted that plants are all right when they are given to us from the nursery or greenhouse, but after a little time they begin to wither. The following precautions should always be observed if success be desired.

Practical Directions.

If possible suitable plants should be obtained. For instance, it is useless to place a delicate Palm whose native home is the West Indies in a large, airy, dry ward, and expect it to do well, when if we wish to grow it in perfection in this country it must be placed in a warm moist stove house or palm house. But fortunately there are many European and North China palms which are hardy enough for this purpose. The leaves should be washed and cleansed thoroughly so that the pores of the leaves may be able to do their work. They should not be allowed to stand in saucers of water, but all the non-flowering plants should be given a good soaking in a bath of water at least once a week, while flowering plants should have it when they require it. There should not be over-watering one day and then forgetfulness to water for many days. If possible, the plants should be hardened off a little before they are brought into the hospital. Perhaps it may be said that it is all very well to lay down these rules, but who has time to carry them out? My reply is that for the little extra care and trouble any lover of flowers will be amply repaid by the plants themselves which really appreciate attention to their wants.

The Best Varieties.

The spring and summer kinds for this sort of work are not very numerous. They include Dracæna, Pandanus, Arabis, Aspidistra, while the best varieties of ferns are Adiantum cuneatum, Asplenium and Polypodium. The best kind of Palms for standing draughts and changes of temperature are the Kentias, the most useful species being Kentia Belmoreana. As to the flowering plants, first of all there is the useful Arum Lily which helps to brighten the dark winter days from Christmas to May. This plant takes a good deal of water, but on no account should it be permitted to become waterlogged or its leaves will drop off after turning a sickly yellow colour if not cut off. Genista or commonly called Cytistus which also comes in about Christmas and gives a rich dash of yellow colour and sweet smell all through the ward needs to be carefully watched in respect to the watering as it so often has a great head of flower for the size of the pot it is in, and will flag very quickly if left without water. Next, the useful plant, the Spirea, claims attention because of its hardiness, but great care should be taken not to allow it to become quite dry or it will collapse and never be the same again; while bulbs can be grown in bowls of sand or cocoanut fibre, or in jars of water in the ward, which will help to amuse the patients as they watch their quick growth. In the summer there will be no lack of pot plants easily obtained, and only needing water when dry, for example, pots of Geranium, Mignonette, Lily of the Valley, etc., which will last longer than cut flowers, and in winter the long flowering Chrysanthemum will be found most useful to stand in the windows and on tables.

Plants at Night.

In conclusion, I should like to say a few words on a subject about which there is much discussion, "whether plants are injurious in a room at night." It has been found out that only under the influence of sunlight do plants absorb carbonic acid gas from the air, through the pores of their leaves, making use of part of it for their own aims, and expiring the refuse as oxygen. If this be the case, then in a room where anyone is ill, it is better to remove the plants at night, because they will only be using up the oxygen which the patient requires, and adding to the amount of carbonic acid gas in the air, no matter how few there may be. Also as the perfume of some strong smelling flowers is very objectionable to people who are ill, it is better to remove them at night as they generally smell strongest at even-tide. If these directions are carried out, and with a little care especially about overwater and underwatering, healthy and beautiful plants will be the result, bringing many memories of the country to the invalid's mind, acting as a stimulant to the worn out body, influencing him to make up his mind to get better and strong again, so that he may in health and strength gaze at the beauties of nature with a thankful heart.

### Garden Party at Gartlock Asylum.

BY A SCOTCH CORRESPONDENT.

To celebrate the Coronation of the King and Queen a garden party was held on Thursday afternoon last week at Gartlock Asylum, near Glasgow. I was favoured with an invitation, and, leaving Buchanan Street Station by special train early in the afternoon, I reached my destination about two o'clock.

Gartlock Asylum is a magnificent building accommodating 600 patients. It is situated in beautifully wooded grounds sloping down to the loch. The guests numbered about 500, and included some 80 children from Barnhill Poorhouse. After a short speech from the Chairman of the Board, the National Anthem was sung, and three cheers were given for the King and Queen. The company then dispersed through the grounds. For an outdoor function the day was perfect. The hawthorn-scented air was warm and still, and the sun shone out at intervals on the gay scene. The children were made happy with games, swings, donkey rides, and that never-failing source of delight, Punch and Judy. It was a pathetic sight to see two boys on crutches, each minus a leg, playing football, and hopping about quite nimbly. Refreshments were served on the lawn, ices being in great demand, so hot was the day. Many of the patients mingled with the visitors in the grounds, and appeared to enjoy the unwonted stir. I was struck by the happy expression on most of the faces, and the affectionate regard the inmates seemed to have for the nurses. One old lady was a source of great amusement. She talked for the most part in rhyme, which came trippingly from her tongue. "I don't smoke, but I like a joke," she told us. To another visitor she said, "I'm not very old, but I've often been told that I'm very bold." There was always a group of admirers round this interesting dame.

Through the kindness of Miss Donald, the matron, I was shown over the asylum and the hospital. In the former building there is a very fine dining-hall, with accommodation for over 400 people. I saw the tables laid for the patients' tea, each of whom was supplied with two large rounds of bread and butter. Some of the tables were covered with white American cloth, and a number with white linen tablecloths. These last are called "family tables," and a patient presides at one end and dispenses "the cup that cheers." There is great rivalry for a place at a "family table." Those who behave best sit there, and receive a *bonne-bouche* every Saturday in the shape of a bun.

The sitting-room is gay with plants and flowers, and ping-pong was being vigorously prosecuted. The large kitchen with its rows of shining pans would delight the eye of a good housewife.

In the hospital only five patients were confined to bed. Surely this speaks volumes for the medical skill and the healthy surroundings of Gartlock.

The pipe band of the Mossbank Industrial School played selections during the afternoon, and step-dancing was engaged in by some of the boys.

At five o'clock tea was served in the Recreation Hall, and the proceedings were enlivened by the asylum string band. The guests left in brakes at 6.30 P.M., many of them carrying away great bunches of blue hyacinth as a reminiscence of a very happy afternoon.

### Everybody's Opinion.

[Correspondence on all subjects is invited, but we cannot in any way be responsible for the opinions expressed by our correspondents. No communication can be entertained if the name and address of the correspondent are not given as a guarantee of good faith, but not necessarily for publication. All correspondents should write on one side of the paper only.]

### THE ROYAL BRITISH NURSES' ASSOCIATION. AND THE MIDWIVES' INSTITUTE.

"Miss J. Wilson, President of the Midwives' Institute," writes: Referring to your account of the meeting of the Royal British Nurses' Association, on June 13th, I beg to say that the Midwives' Institute did not elect or send a representative to the conference, for the simple reason that the Institute received no communication on the subject, nor were we asked to send a representative. The fact that Miss Oldham happens to be a member of a Council of the R.B.N.A., and is also a member of the Council of the Midwives' Institute, does not alter the fact that she did not attend the conference as a *representative* of the Midwives' Institute, nor did she understand that she was invited by the executive committee of the R.B.N.A. to attend in that capacity.

### PRIVATE NURSING HOMES.

"H." writes: May I suggest not only the expediency but the urgent necessity for private "nursing homes" to be thoroughly investigated and subjected by Government to a strict and regular supervision? The persons who run these establishments seem, and generally are, both ignorant of the work required, and perfectly callous as to the comfort of the unfortunate invalids who, either through the advice of unconscientious medical men, or by some equally unreliable means, become helpless inmates for weeks and sometimes months of what are mere money-grabbing concerns, which are a disgrace to such a city as London, and such a country as England! These places are for the most part dirty, noisy, and ill-conditioned; the majority of their so-called nurses are untrained, inconsequent girls, who experiment for the sake of their own ends on suffering humanity, or waste their time away from the patients committed to their charge; and are under either circumstances utterly unfitted for the care of serious cases. The food is usually atrocious, and the general neglect most reprehensible. For this the fees are exorbitant, and the evil is incalculable.

### THE FOOD QUESTION.

"E. S. E." writes: Poor "M. A. B." I think she must be feeling very ill and needs a holiday and tonic, and would advise her to take both. Would it not be better if she referred this grievance to her matron instead of publishing it, and failing any good results therefrom placed the grievance before the committee. But I don't think the matron would turn a deaf ear to such a complaint if there is cause for it. I remember once complaining to my matron about the food, and will never forget her anger; but she certainly looked into it, and the result was highly satisfactory. I think if matrons were told when things go wrong they would do their best to set them right. Certainly cold meat, bread, and water does not sound very appetising, but tea, and bread and butter is very good for tea, of course. Pork scarcely warmed through and jaundiced potatoes are not nice, but perhaps this happened just on one day when the cook was off duty, and if a good dinner is provided six days in the week we must allow for accidents. I have never known "stewed tea" to be provided in the morning; I did not think that the maids had time to do it for breakfast. I have been nursing nearly eight years, and have always found that nurses who are ladies rarely complain, while those taken from the servant class are always grumbling.

### THE INSTANT DISMISSAL OF PROBATIONERS.

"Marcus" writes: Being myself a probationer, and having had more than two years' training in my present hospital, I feel quite competent to answer the—may I say absurd?—letter written by "Justice with Mercy." On behalf of matrons and sisters in general, I can say very little it having been my pleasure to know only those under whom I am training. But I cannot help thinking that if "Justice with Mercy" had been fortunate enough to have fallen in with this school, her letter would never have been written. The more than two years spent within these walls are among the happiest of my life, in spite of many scoldings and lectures to which all would-be nurses are heirs. Our matron has always found her punishment of confiscating a day off duty quite adequate to the offence. As for whipping a probationer! Was it not Matthew Arnold, who said, when writing about schoolboys and their punishments, "Only the most depraved and hopeless lads should be flogged"? Surely then, "Justice with Mercy" must have fallen very low in her own estimation to have expected a whipping at any period of her training. I will not criticise the sister who performed the shaking. Not one of the four under whom I serve would have stooped to the action. When "Justice with Mercy" becomes a sister where does she intend looking for someone to whip? Methinks most women of 21 or 22 years have long since put behind them all visions of canes and, generally, intentional disobedience. May all the pros under her commit only first offences, for I am sure a day in bed will be quite enjoyable. Is there a nurse who does not love her day in bed? Justice, as I read her, is certainly not tempered with loyalty, and I fail to discover any mercy to which she can lay claim.

### Appointments.

[No charge is made for announcements under this head, and we are always glad to receive, and publish, appointments. But it is essential that in all cases the school of training should be given.]

Bridgwater Infirmary.—Miss M. Shorto and Miss E. Slaughter have been appointed charge nurses. They were both trained, for three years, at the East Suffolk Hospital, Ipswich; and Miss Slaughter has also had two years' training at the Cottage Hospital, Woking.

Kingston-upon-Hull Sanatorium.—Miss Susan A. Musson has been appointed sister-in-charge. She was trained at Liverpool Royal Infirmary for three years, and has since been charge nurse and night sister—taking the duties of home sister—at Hull Royal Infirmary.

Morecambe Cottage Hospital.—Miss Annie Beaumont has been appointed nurse-matron. She was trained at the Royal Infirmary, Sheffield, and has since been successively staff nurse, sister to children's medical and surgical wards and sister to male accident wards, in the same institution.

Newark-on-Trent Infirmary.—Miss Loveday Gaved-Wills has been appointed Lady Superintendent. She was trained at the Devon and East Cornwall Hospital, Plymouth, where she was afterwards staff nurse and on the private nursing staff. She has since been charge nurse at St. Peter's Hospital, Covent Garden, London, charge nurse at the Infirmary, Newport, Mon., night sister at the Devon and East Cornwall Hospital, night superintendent and day sister at the West London Hospital, Hammersmith, W.

### To Nurses.

**We invite contributions from any of our readers, and shall be glad to pay for "Notes on News from the Nursing World," or for articles describing nursing experiences, or dealing with any nursing question from an original point of view. The minimum payment for contributions is 5s., but we welcome interesting contributions of a column, or a page, in length. It may be added that notices of appointments, entertainments, presentations, and deaths are not paid for, but that we are always glad to receive them. All rejected manuscripts are returned in due course, and all payments for manuscripts used are made as early as possible after the beginning of each quarter.**

### A Book and its Story.

"THE SOUND OF A VOICE THAT IS STILL."

No suggestion is made in the little volume before us* as to the circumstances under which the papers, that originally appeared in *The Pilot*, were written, and a brief note of explanation would have been welcome; but a pathetic interest is attached to their appearance in the present form, as being the last words of the author, who died while the book was in the press. The first edition was sold out at once and a second one called for, which proves that there is always a public waiting for the voice of a writer like Michael Fairlees, gifted with the power of expression of those feelings which, for them, lie too deep for words. With masculine breadth of view, the author combines feminine insight and grace of expression. She was one to whom Nature and her fellow-men were a moving source of inspiration, and she dwelt under the shadow of the land that is very far off, waiting trustingly for the summons to enter into the rest for which her soul, by suffering, was being prepared. The perfect literary form of the papers reveals a rare spirituality of mind and much culture and refinement of imagination. The book is divided into three sections, from the first of which, "The Roadmender," the title is taken. "Out of the Shadow" and "At the White Gate," are the two that follow. From his heap of stones by the roadside the roadmender saw some things that would not be visible to all eyes. "All day I sit on a stretch of grass under a high hedge of saplings and a tangle of traveller's joy, woodbine, sweetbriar, and late roses. Opposite me is a white gate seldom used, if one may judge from the trail of honeysuckle growing tranquilly along it; I know now that whenever and wherever I die, my soul will pass out through this white gate; and then, thank God, I shall not need to undo that trail. In youth we discussed our ideals freely; I wonder how many besides myself have attained, or would understand my attainings. After all, what do we ask of life, here or indeed hereafter, but leave to serve, to live in commune with our fellow-men and with ourselves; and from the lap of earth look up into the face of God? All these gifts are mine as I sit by the winding white road and serve the footsteps of my fellows." The roadmender has his word with passers-by, from the tramp to the pretty girl who borrows string from him to tie up the dressguard of her bicycle. "When I had tied it for her she looked at me, at my worn dusty clothes and burnt face; and then she took a Niphetos rose from her belt and laid it shyly in my dirty disfigured palm. I bared my head, and stood hat in hand looking after her as she rode away up the hill. Then I took my treasure and put it in a nest of cool dewy grass under the hedge."

The character vignettes of the humble people who pass to and fro are touching and life-like, and outlined with delicate perception. The following one is of an old man, a typical countryman, too old to work, too poor to add his mite to household expenses of the son who had sheltered him in his own cottage until it was no longer possible as little mouths increased that had to be fed. So, "I'm tramping my way to N—— to the House; it's a 'ard pinch, leavin' the little ones. . . . . I'm eighty-four," he went on, "and terrible bad with the rheumatics in my chest. Maybe it'll not be long before the Lord remembers me." Then comes a little scene, a touch of nature which makes the whole world kin. The roadmender is guarding a little child for whom the road was a weary way on that hot afternoon. She had been left by her grandparents as they passed, to be called for later. "Ah!" he said, "I've left a little darlin' like this at 'ome." The child crept close and put a sticky little hand confidingly into the tired old palm. The two looked strangely alike, for the world seems much the same to those who leave it behind as to those who have taken the first step on its circular pathway." How true also is the picture of the widow with whom the roadmender lodged. "She had been deaf for the last 20 years and she speaks in the strained high voice which protests against her own infirmity. Her eyes have the pathetic look of those who search in silence. For many years she lived alone with her son, who laboured on a farm two miles away. He met his death in rescuing a cart-horse from its burning stable. . . . Of death she has no fears, for in the long chest in the kitchen lie a web of coarse white linen, two pennies covered with the same, to keep down tired eyelids, decent white stockings, and a white cotton sun-bonnet—a decorous death-suit truly—and enough money in the little bag for self-respecting burial.

Nothing could be more charming than the scene of Sabbath peace pictured in the following little sketch:—"On Sundays my feet take ever the same way. First my temple service, and then five miles tramp over the tender, dewy fields, with their ineffable earthy smell, until I reach the little church at the foot of the grey-green down. Here, every Sunday, a young priest from a neighbouring village says Mass for the tiny hamlet, where all are very old or very young, for the heyday of life has no part under the long shadow of the hills, but is away at sea or in service. There is a beautiful seemliness in the extreme youth of the priest who serves these aged children of God. He bends to communicate them with the reverent tenderness of a son, and reads with the careful intonation of far-seeing age. To the old people he is the son of their old age, God-sent to guide their tottering footsteps along the highway of foolish wayfarers; and he, with his youth and strength, wishes no better task." The author has a word to say on the now defunct subject of the writer of "An Englishwoman's Love Letters." "As for the authorship, there is a woman's influence, an artist's poorly concealed bias in the foreign letters, and for the rest a man's blunders—so much easier to see in another than to avoid oneself—writ large from cover to cover. . . . Mrs. Meynell! cries one reviewer triumphantly. Nay, the saints be good enough to us, what has Mrs. Meynell in common with the Englishwoman's language, style, or most unconvincing passion? Men can write as from a woman's heart when they are minded to do so in desperate earnestness. There is Clarissa Harlowe, and Stevenson's Kirstie, and many more to prove it; but when a man writes as the author of the 'Love Letters' writes, I feel as did the painter (of a certain stencil frieze) that pattern-making has gone too far, and included that which like the grass should be spared such a convention." With an extract which is itself a poem in prose, taken from some reflections on the passing of a funeral procession, we must conclude our notice of this lovely little book. A book to have by one and to keep. "To the large majority death is Pluto, king of the dark unknown, whence no traveller returns, rather than Azrael, brother and friend, lord of the mansion of life. . . . When the hour strikes he comes, very gently, very tenderly, if we will but have it so, folds the tired hands together, takes the way-worn feet in his broad palm, and lifting us in his wonderful arms he bears us swiftly down the valley across the waters of remembrance."

* "The Roadmender." By Michael Fairlees. 1 vol. 2s. 6d. net. London: Duckworth and Co.

### Echoes from the Outside World.

### The Queen's Tea.

IT is announced that the Bishop of London is arranging, as far as possible, to enlarge the scope of the Queen's Coronation Tea to general servants. As originally proposed, only those connected with three organisations for domestic servants were to be allowed to participate, but the lines of demarcation have now been widened, and in addition to girls who are members of Church of England societies, Wesleyans and Romanists are to share in the treat. Some hundreds of the applications received by letter from those who are in touch with no special society are also to be considered, and the writers included if practicable.

### The Coronation.

UGANDA is represented at the Coronation, by Apolo Kagwa Katekiro, the Prime Minister of that country. When asked, through an interpreter, what had most impressed him upon his arrival in England, he replied, "We think the ladies so nice. We had seen pictures of them and their dresses, but we used to think those were fancy pictures."

A "Coronation Concert" was given last Saturday afternoon at the Albert Hall. Madame Albani contributed three items, including "Robin Adair," and in response to an encore she gave "Home Sweet Home." The selection of the fare offered to the concert-goers had evidently been influenced by the Coronation. A march by Mr. Coleridge-Taylor, illustrative of "Ethiopia Saluting the Colours," written for the occasion, was performed by the orchestra, accompanied by the organ. "Great, and Still Greater," composed by Mr. A. Randegger, jun., was given by Mr. Kennerly Rumford, accompanied by the male choir; and Madame Clara Butt sang for the first time a new song entitled "Land of Hope and Glory," which roused the patriotism and the enthusiasm of the audience. A large number of soldiers in khaki and other uniforms were present, and several ladies in the native dress of their respective countries were conspicuous by their bright and handsome costumes.

### Lord Kitchener on his Way Home.

LORD KITCHENER left Capetown on Monday and is expected in England about July 12th. Before his departure from Johannesburg he was entertained at a great banquet, which is described as one of the most remarkable celebrations of the kind in South Africa. Besides Lord Milner and the leading officers of the Army, practically all the leading citizens were present to do honour to the guest of the evening. Lord Milner paid a high tribute to Lord Kitchener and the Army, at the conclusion of which the audience cheered vociferously for several minutes; and then Lord Kitchener delivered a speech of considerable length, in which he dwelt on the lessons of the war, urged his hearers to keep their horses and rifles ready and their bodies physically fit, so that they might be prepared at any time to take their due part in the great Empire which united them all, and bade them remember that the advent of the happy time of complete reconciliation greatly depended upon the way in which the Boers were treated. It is said that, if his own personal wishes were consulted, Lord Kitchener would prefer to land in England quite unobtrusively, take a hansom cab at the railway station, and report himself at the War Office the same afternoon. But he will probably have to submit to a great public reception.

### Other Returning Generals.

GENERAL FRENCH—who, with General Walter Kitchener and Colonel McCalmont, is on his way to England—before leaving Middelburg publicly thanked the inhabitants, and especially the Dutch, for their loyalty, and expressed the hope that even the rebels would be met half way in a friendly spirit, for, he said, "one must not overlook the powerful influence exerted by ties of blood and feelings of sympathy." Lord Methuen is also fast nearing his native shore. Hearing that Sir John Goldney was chairman of a committee to welcome him back, he wrote him a letter from Johannesburg Hospital to express the hope that Corsham would not think him forgetful of the happy relations which have always existed between the people and his family if he asked as a personal favour that he might be permitted to return to his home "as quietly as he left it two and a half years ago." Lord Methuen continued: "You can understand that one does not easily forget the many friends and comrades lost in this war, and out of respect to them I earnestly desire to avoid any public reception after my return to England."

### Foreign.

THE death of the King of Saxony was by no means unexpected, as he had been lying in a dangerous condition for two or three weeks. He was 74 years of age, and in his youth he received a thoroughly military education, becoming a lieutenant in the Army at the age of 15. He fought on the side of Austria in the disastrous battle of Sadowa, and also took a conspicuous part in the Franco-Prussian war. On the investment of Metz he received the command of the Army of the Maas, was present at the capitulation of Sedan, and held the right bank of the Seine during the seige of Paris. After the war he was appointed a Field Marshal by the German Emperor. He was married to Princess Carola of the old Royal Dynasty of Sweden, but he had no children. The day before his death was the forty-ninth anniversary of his marriage. During the morning his Majesty sent for a rose, and after gazing at it for some time he motioned the Queen to his bedside. He was very weak and in great pain, but a wonderful look came into his eyes as, without saying a word, he presented the flower to his loved wife. The Queen bore up bravely for a moment, and then burst into tears. Prince George, who succeeds the King, is 70 years of age. The ball which was to have taken place at Windsor Castle on Thursday last week was abandoned upon receipt of the news at the English Court, and the King has commanded Court mourning for three weeks, the order to be suspended during the Coronation festivities.

### The Truth about St. Pierre.

M. JEAN HESS, the well-known explorer, who has just returned from St. Martinique, describes in detail the terrible catastrophe at St. Pierre. He also explodes certain widely accepted legends, including the story of a unique survivor having been found in the prison, where he was confined on a charge of murder. M. Hess affirms that not a soul in the city escaped. Consequently there were no injured to succour. The last telegram despatches from St. Pierre which were received at Fort de France were published on Saturday. The final intelligible communication consisted of the letters O. K., equivalent to "all right." Then followed an indistinct noise, and a final signal which took the form of a cross.

### Philanthropic.

ON Saturday Lord Meath opened a home for epileptic children in connection with Lingfield Farm Colony, a social undertaking for the assistance of men of the submerged tenth. It is the gift of Mrs. Ruston, of Lincoln, and bears her name. The colony was started some years ago, and it speedily developed as an important side of its work homes for epileptic children. The Ruston Home, which was erected at a cost of £2,800, is the second. The work has been carried out on lines suggested by the previous experience of the Christian Union with epileptic children, who are recommended to them by different Boards of Guardians under the direct sanction of the Local Government Board. It is stated that out of 65 children 25 per cent. have been practically cured.

### For Reading to the Sick.

ST. PETER'S DAY.

I FOLLOWED Thee, my God, I followed Thee
To see the end:
I turned back flying from Gethsemane,
Turned back on flying steps to see
Thy Face, my God, my Friend.

Like as the hart the water-brooks I Thee
Desire, my hands
I stretch to Thee; O kind Lord, pity me:
Lord, I have wept, wept bitterly,
I driest of dry lands.

Scarce in Thy throne and kingdom; yet with Thee
In shame, in loss,
In Thy forsaking, in Thine agony:
Love crucified, behold even me,
Me also bear Thy cross.

*C. Rossetti.*

At Gethsemane, St. Peter fled with all the rest, and then, stung with remorse, went back "to see the end," and joined the company of soldiers and maidservants. "Thou also wast in the garden with Him?" said one of the bystanders. "I know not the man," is the prompt reply. And then the cock crew; and while our Lord is led bound across the court, He meets St. Peter.

"The Lord *turned*"—it was a deliberate act—"and looked on Peter." We know how the eyes can speak more eloquently often than the lips. The eyes of a friend, the eyes of the dying, they say so much. So, too, that look of our Lord's conveyed to St. Peter a message of gentle reproach, yet of full forgiveness, of unchanging love. It was as though our Lord said: "Did I not tell you so? Watch and pray lest you enter into temptation."

St. Peter never forgot that look of his Master, and it is recorded for our instruction. Our Lord often looks on us; indeed He is always doing so, but we may feel it more at one time than at another. He looks at us with eyes of welcome in Holy Communion, with eyes of joy and compassion, with eyes of hope as He offers us our daily graces, with eyes of congratulation when we have done well, with eyes of friendship when we have sinned; and by His sick and suffering children our Lord is every standing, watching their pain and need, and the look of His eyes seems to say: "I am with you in tribulation; I see it all; it is all noted down in the book of life." This thought may help you, especially towards evening, after a long day, as you raise your eyes to heaven and meet our Lord's cheering look, to tell you all is well, that he is satisfied; and tears may come to your eyes, but they will be tears of joy and peace.—*Anon.*

O Jesu, gone so far apart
Only my heart can follow Thee,
That look which pierced St. Peter's heart
Turn now on me.

Thou who dost search me thro' and thro'
And mark the crooked ways I went,
Look on me, Lord, and make me too
Thy penitent.—*C. Rossetti.*

### Notes and Queries.

**The Editor is always willing to answer in this column, without any fee, all reasonable questions, as soon as possible.**
**But the following rules must be carefully observed:—**
**1. Every communication must be accompanied by the name and address of the writer.**
**2. The question must always bear upon nursing, directly or indirectly.**
**If an answer is required by letter a fee of half-a-crown must be enclosed with the note containing the inquiry, and we cannot undertake to forward letters addressed to correspondents making inquiries. It is therefore requested that our readers will not enclose either a stamp or a stamped envelope.**

*Mental Nursing.*

(101) I am 24, and am anxious to be trained as a mental nurse, preferably at Wakefield. Would you kindly give me the correct address, and tell me if there is any premium to pay. Am I too old or too young?—*E. W.*

Apply the West Riding Asylum, Wakefield, Yorks., for all particulars. You are a very suitable age.

1. What length of training must one undergo in order to become a qualified mental nurse, and what are the best institutions at which to apply? 2. Is the holder of a St. John's Ambulance medallion able to take the post of a nurse? or 3. Should she take a course of training? 4 Are there any hospitals, public or private, which give one year's training upon the payment of a premium? 5. Are there any homes for training district nurses?—*Edna.*

1. The best schools for mental training require their probationers to earn the certificate of the Medico-Psychological Society, for which the training occupies a course of three years. A list of the asylums preparing for this examination is given in the "Nursing Profession: How and Where to Train." 2. No. 3. Yes. 4. Yes; see the nurses' manual referred to above. 5. The Queen Victoria's Jubilee Institute for Nurses trains nurses for district work. Apply, the General Superintendent, St. Katherine's Precincts, Regent's Park, N.W.

*Vaccination.*

(102) Will you kindly inform me if an unsuccessful vaccination indicates that the person vaccinated would not contract small-pox if exposed to infection? I have just been vaccinated and, beyond a slight irritation, there has been no result.—*Inquirer.*

There are so many things that may prevent the vaccination taking that it would be safer to be re-vaccinated if you are going to be exposed to infection.

*Up-Country Nursing Association.*

(103) Will you kindly tell me to whom I should apply for particulars of the Up-country Nursing Association?—*F. R.*

Apply to the Hon. Secretary, H. M. Bird, Esq., Dalkeith House, Cambridge Park, Twickenham.

*Small-pox.*

(104) Will you kindly tell me in which number of THE HOSPITAL the answer of the first prize winner on small-pox was given?—*L. L.*

In that of December 21st, 1901.

*Fœtal Head and Pelvis.*

(105) Can you kindly suggest any place where I might get a fœtal head and pelvis to practise on a few times? I have just come over from India to take my L.O.S., and I wish to go through my positions again.—*G. C.*

Write to the Secretary of the Midwives' Institute, 12 Buckingham Street, Strand, W.C., as she would probably be able to help you.

*Sanitary Inspectors.*

(106) Will you kindly give me any information regarding the examination for lady sanitary inspectors, and also say if one stands a good chance of getting a post if one passes the required examination.—*An Inquirer.*

Write for particulars to the Sanitary Institute, Margaret Street, London, W. There may be some little difficulty in getting employment at first, but there are fair openings for suitable persons.

**Standard Nursing Manuals.**

"The Nursing Profession: How and Where to Train." 2s. net; post free 2s. 4d.
"Art of Massage." (Second Edition.) 6s.
"Elementary Physiology for Nurses." 2s.
"Elementary Anatomy and Surgery for Nurses." 2s. 6d.
"Practical Handbook of Midwifery." 6s.
"Surgical Ward Work and Nursing." Revised Edition. 3s. 6d. net; post free 3s. 10d.
"Mental Nursing." 1s.
"Art of Feeding the Invalid." 1s. 6d.
All these are published by the SCIENTIFIC PRESS, Ltd, and may be obtained through any bookseller or direct from the publisher, 28 and 29 Southampton Street, London, W.C.

### Travel Notes.

By Our Travelling Correspondent.

### CII.—WHAT WE CAN SEE FOR FIVE POUNDS.

### Mechlin.

I must begin by saying that trips to Belgium are only interesting to those whose tastes lie in the ecclesiastical and domestic architecture of the past. The beautiful old towns of Flanders are a perpetual joy to the artist, the photographer, the antiquarian, and the historian, but of scenery there is nothing worth speaking of; the country stretching between these old-world cities is all of much the same character, verdant pastures, very few trees, plenty of canals, and a large proportion of windmills. True, down in the Ardennes, the landscape is beautiful, but in Belgium, pure and simple, there is a flatness and monotony far from pleasing.

### The Cheapness of Belgium.

Who does not appreciate this delightful peculiarity? It is a pleasing distinction which is fast disappearing before the devouring hordes of tourists that flood the Continent in the present day. You may comfortably spend a week, or indeed a fortnight at Mechlin for the sum of £5 if you go the cheap way from Tower Bridge. By this route the return ticket is only £1 second class, and you can get good accommodation in Mechlin for 6f. per day. The other route, which is a trifle dearer, is by Harwich and Antwerp, and comes to £1 7s. second return.

### A Good Centre for Excursions.

Antwerp, Brussels, Louvain, Alost and Ghent are all within a day's excursion from Mechlin, so that, with the exception of Bruges and Courtrai and perhaps Oudenarde you could in this cheap trip see the most interesting parts of Old Flanders. With a little management you can visit Antwerp on your way, if you take the Harwich route, and if not, the expense of the journey is very trifling, not more than 2s. third return. Third class is quite clean and comfortable: I always travel so if I can make hours fit, it is much more amusing, considerably cheaper, and the company generally most kind and polite. I think I should recommend you to the Hôtel La Cour de Beffer; ask for rooms on the third floor and propose pension terms at 6f. per day. At my first visit I stayed at a charming old-fashioned hotel which, I regret to say, is now closed. It had been a convent and was most picturesque.

### The Cathedral.

This is usually one's first point in most continental towns. Look up at the clock-face on the tower and try to realise that it is 40 feet across. The church is dedicated to St. Rombold, and dates from the twelfth century, but almost the whole of the first church was burnt in the middle of the fourteenth, so that the present building was chiefly built in the end of the fourteenth and beginning of the fifteenth centuries. In the south transept is a most truly beautiful altar-piece of Van Dyck, representing the Crucifixion. I was never weary of studying it; though, alas! being covered, it means a fee every time. The pulpit, representing the conversion of St. Paul, is of the usual Flemish type, perhaps more wonderful than beautiful. The stained glass is modern and of no great merit.

### The Church of Notre Dame.

This is chiefly remarkable for Rubens' world-famous "Miraculous Draught of Fishes," so familiar to us from cartoons, prints, oleographs, etc. It was painted for the guild of fishers, and Rubens received for it a paltry sum—under £100. The Sacristan lives opposite the chief Portal; you must remember the churches are generally closed for about an hour and a-half at mid-day. Very near is the only remaining bit of the old fortifications—the Porte de Bruxelles, with double towers; this is yearly threatened with destruction, perhaps it has already fallen.

### Mechlin is a Town of Leisure.

It is wonderfully sleepy, and except in the market place there seems very little liveliness. Some 15 years ago I made delightfully cheap purchases of peasant jewellery in a quaint little shop officially dedicated to the sale of bacon and cheese. Exquisite examples of delicate gold and silver work hung in the window, in company with toothsome sausages and grinning pigs' faces. The old proprietor (knowing nothing of the art of curio selling) placed the gems in his scale, from which he displaced a most objectionable piece of pork, and sold them by weight. One treasure that I secured was a most beautifully chased and embossed gold clasp of a barrel shape to secure a necklace. I had not money enough to pay for it, but the old man was quite willing that I should take it and pay the next day. This I would not do, but ran home for more, arriving at his strange emporium, breathless but not later than 8 o'clock. He was putting up his shutters, and would by no means listen. "To-morrow, to-morrow," he ejaculated placidly. "Plenty of time, plenty of time!"

That old man was the very impersonation of elegant leisure. We visited him many times and he regarded us with good-natured indulgence. We always had to unhook the articles ourselves if they were not within reach of his arm, as he sat in a species of Windsor chair behind his counter; to rise and fetch anything was quite too much exertion. He looked mildly astonished at our energy, gazing benevolently at us through silver-rimmed spectacles, then ejaculated, "And you are English? Well! well!" as if at last pleased to have seen with his own eyes, specimens of a strange and barbarous race of whose existence he had heard, but hardly believed in.

### Domestic Architecture.

Mechlin is very rich in this and also in fine municipal buildings. The Grande Place is as fine as any in Belgium I think, unless it might be Ypres, although its town hall is far less beautiful than those of Brussels or Louvain. There is the sixteenth century Palais de Justice, and next to it the old cloth hall; but what I like best is the Schepenen-Huis, it has an outside stone staircase and a very steep gabled roof with turrets at the corners. It is now used as the museum. I secured a sketch of it by moonlight; it was a great deal of trouble, but it was worth it. On the picturesque Quai au Sel are some very fine houses, three which stand together are Le Maison du Paradis and Le Maison de St. Joseph, separated by a third called Le Maison du Diable. This last is in very dark timber and is much the handsomest of the three. Next week I must tell you something of those places which can be seen easily from Mechlin.

### TRAVEL NOTES AND QUERIES.

Cheap Holiday in Italy (Undine).—It all depends on what you call cheap. In most Italian towns, if you know how to manage, you can live and board for 6 lire, which are 5s.; but even this comes to 35s. per week without extras. If you are meaning to make a long stay, however, in such towns as Perugia, Siena, or even Florence or Venice, you can usually make an arrangement for 25s. to 27s. per week. A still cheaper way is to take a single room and cater for yourself; but this is not to be thought of unless you can understand and speak Italian tolerably well; without that accomplishment do not attempt it. I should not advise Orvieto for your purpose, rather try Siena or Perugia. Ravenna has the name, whether truly or not I know not, of being somewhat unhealthy for those not acclimatised.

Bruges for Sketching (Trilby).—Yes, you could stay there a month very comfortably for the sum you mention; 6 francs will board you very well, and it is in no way a dear place. I like best the Hôtel Panier d'Or in the market place. I think they would meet your terms there, and they are most kind and attentive people. The place you ask about must be Damme. I think, two or three miles from Bruges on the Sluis route. A short time since one went there by barge, but I believe now a steamboat has invaded the canal. There is not very much to be seen, and since the quaint journey by barge is abolished I do not think it is worth your consideration.

## Figures and Tables

**Fig. 47. f1:**
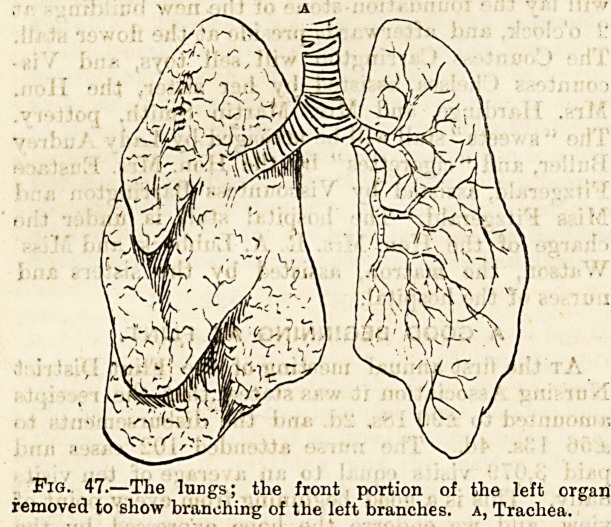


**Fig. 48. Fig. 49. f2:**